# Simultaneous PSI-Based Orthognathic and PEEK Bone Augmentation Surgery Leads to Improved Symmetric Facial Appearance in Craniofacial Malformations

**DOI:** 10.3390/jpm12101653

**Published:** 2022-10-05

**Authors:** Valentin Kerkfeld, Lara Schorn, Rita Depprich, Julian Lommen, Max Wilkat, Norbert Kübler, Majeed Rana, Ulrich Meyer

**Affiliations:** 1 Department of Oral-, Maxillofacial and Facial Plastic Surgery, University Hospital Düsseldorf, Moorenstraße 5, 40225 Düsseldorf, Germany; 2 Center for Jaw-, Face- and Skull Malformations, Schorlemerstraße 26, 48143 Münster, Germany

**Keywords:** orthognathic surgery, patient-specific implants, PEEK, craniofacial reconstruction, craniofacial malformation, digital workflow

## Abstract

(1) The aim of the present study was to compare the outcome of facial symmetry after simultaneous digitally planned patient-specific implant (PSI-) based orthognathic surgery and polyether ether ketone (PEEK) bone augmentation in patients with craniofacial malformations. (2) To evaluate the outcome of the two different surgical approaches (conventional PSI-based orthognathic surgery versus simultaneous PSI-based orthognathic surgery with PEEK bone augmentation), a comparison of five different groups with a combination of the parameters (A) with vs. without laterognathia, (B) syndromic vs. non-syndromic, and (C) surgery with vs. without PEEK bone augmentation was conducted. The digital workflow comprised cone beam CT (CBCT) scans and virtual surgery planning for all patients in order to produce patient specific cutting guides and osteosynthesis plates. Additionally, deformed skulls were superimposed by a non-deformed skull and/or the healthy side was mirrored to produce PSI PEEK implants for augmentation. Retrospective analyses included posterior–anterior conventional radiographs as well as en face photographs taken before and nine months after surgery. (3) Simultaneous orthognathic surgery with PEEK bone augmentation significantly improves facial symmetry compared to conventional orthognathic surgery (6.5%P (3.2–9.8%P) (*p* = 0.001). (4) PSI-based orthognathic surgery led to improved horizontal bone alignment in all patients. Simultaneous PEEK bone augmentation enhanced facial symmetry even in patients with syndrome-related underdevelopment of both soft and hard tissues.

## 1. Introduction

Orthognathic craniofacial surgery aims to create a functional and symmetric bone and soft tissue appearance. Symmetry is defined as a perfect match between the right and left side of the face [1]. In many patients, craniofacial deformities are accompanied by malocclusion necessitating orthognathic surgery to the facial skeleton [2]. Numerous studies have reported changes of hard and soft tissues in patients after orthognathic surgery [3,4,5]. However, the reconstruction of bone symmetry described by these studies rarely provided sufficient soft tissue symmetry. This effect is partly due to inevitable surgical detachment of facial muscles in cases with extreme bone deformities. Hence, extensive soft tissue volume deficiencies between the right and left side of the face occur [6]. This negative effect in the therapy of craniofacial malformations has recently been countered using polyether ether ketone (PEEK) implants for bone augmentation [7]. Since the invention of 3D printing in 1984 [8], computer-aided design (CAD) and computer-aided manufacturing (CAM) have opened up new opportunities for modern medicine to improve the surgical outcome for each individual patient [9,10]. Many studies have already demonstrated the higher accuracy of CAD/CAM technology for intraoperative splints in orthognathic surgery [11,12,13,14,15,16]. Even more, patient-specific implants (PSI) have revolutionized orthognathic surgery in recent years by allowing accurate preoperative planning and by assisting the surgeon in proper positioning [17,18,19]. The advantages of PSI-based orthognathic surgery are well described, and the procedure has been proven for many years [20,21,22,23]. Furthermore, augmentative procedures have been established in which PEEK bone implants can be used to adequately replace bone defects or malpositions [24,25,26,27,28,29]. In recent years, the great benefit of PEEK bone augmentations for the treatment of facial asymmetries has been discovered. This has made it possible to achieve further improvements in facial symmetries after orthognathic surgery [18,27,30,31]. In particular, soft tissue symmetry is significantly improved [19]. In contrast to autologous replacement therapies, PEEK augmentations benefit from the lack of donor site morbidity and infinite availability [32]. However, PEEK augmentations also come with several disadvantages as patients must undergo another operation (two-stage approach [33]) with all its surgical and anesthesiological risks [34]. In addition, overall therapy time is prolonged, which is associated with poorer patient compliance and satisfaction [35]. Until now, patients with laterognathy first receive (PSI-based) orthognathic surgery, followed by correction of any hard and soft tissue asymmetry with PEEK bone implants in a second approach. No study has yet investigated the clinical benefits of a simultaneous PSI-based orthognathic and PEEK bone augmentation approach in craniofacial surgery to achieve accurate symmetry.

Patients with non-laterognathic dysgnathia and symmetrical craniofacial malformations (e.g., Apert or Crouzon syndrome) represent a simpler group of patients in whom symmetry can be achieved after surgery. Patients with laterognathic conditions, whether syndromic or non-syndromic, are a much more complicated patient group in terms of establishing symmetry.

In terms of the surgical precision it should be noted that occlusal rehabilitation requires a precision of 20–40 µm [36] whereas orthognathic surgery solely demands an accuracy of 1.0 mm in linear distance deviation and 2–4° angular deviation as occlusal fine-tuning is achieved postoperatively through orthodontic treatment [11]. In regards to facial symmetry 1.0–2.0 mm deviation is hardly visible, likely due to the fact that no viscerocranium is perfectly symmetrical by nature [37].

The question arises as to whether modern surgical planning and execution tools can achieve such symmetry in patients with laterognathy including those with severe craniofacial malformations. Therefore, the aim of the present study was to evaluate whether a standardized protocol of PSI-based orthognathic surgery with simultaneous PEEK bone augmentation improves facial symmetry of the soft and hard tissue.

## 2. Materials and Methods

This retrospective study is a cephalometric (p.a. radiography) and photographic (en face) analysis of patients with various craniofacial malformations (syndromic and non-syndromic as well as with and without laterognathy) who underwent different surgical therapies (conventional PSI-based orthognathic surgery vs. PSI-based orthognathic surgery combined with simultaneous PEEK bone augmentation).

### 2.1. Patients

The study included 30 patients in five groups (Table 1). Group 1 represents the control group, comprising only patients without laterognathy or syndromes. Groups 2 and 3 included patients with laterognathy who underwent conventional PSI-based orthognathic surgery, while groups 4 and 5 underwent PEEK bone augmentation simultaneously to conventional orthognathic surgery. Patients in groups 3 and 5 had craniofacial malformations due to congenital syndromes such as (bilateral) craniosynostosis (group 3) and (unilateral) branchial arch disease (group 5).

All patients were treated by the same craniomaxillofacial surgeon.

### 2.2. Planning Protocol

**Figure 1:**
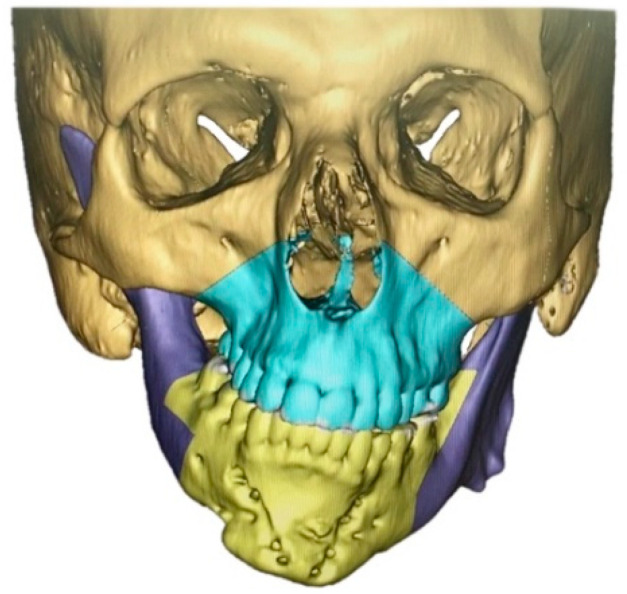
Preoperative virtual planning. Baseline situation as identified by CBCT. Color-coded: maxilla (turquoise), corpus mandibulae (yellow), and rami mandibulae (purple).

**Figure 2:**
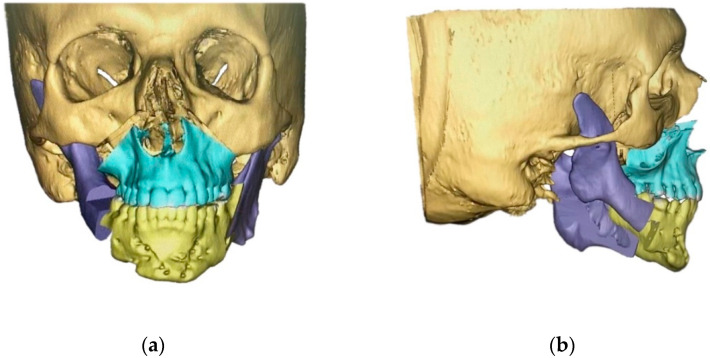
Preoperative virtual planning. The surgical procedure is planned virtually in advance and the three jaw components (maxilla (turquoise), corpus mandibulae (yellow) and rami mandibulae (purple)) are aligned three-dimensionally in position, tilt, and rotation. (**a**) Frontal view, (**b**) lateral view.

As part of the digital workflow, all patients received a CBCT scan. Virtual orthognathic surgery (Materialise GmbH, Munich, Germany) was then performed for all groups. Patient-specific osteosynthesis plates (Synthes GmbH, Oberdorf, Switzerland) and surgical cutting guides were fabricated based on virtual planning (Figure 1 and Figure 2), followed by surgery.

**Figure 3:**
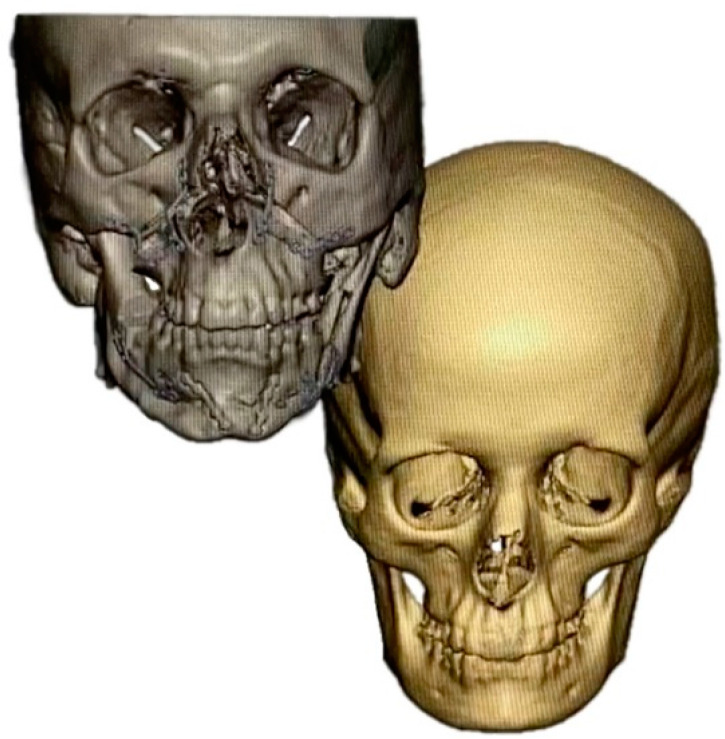
Superimposition strategy. Patient skull after virtual planning of PSI-based orthognathic surgery (gray) and standard skull (beige).

**Figure 4:**
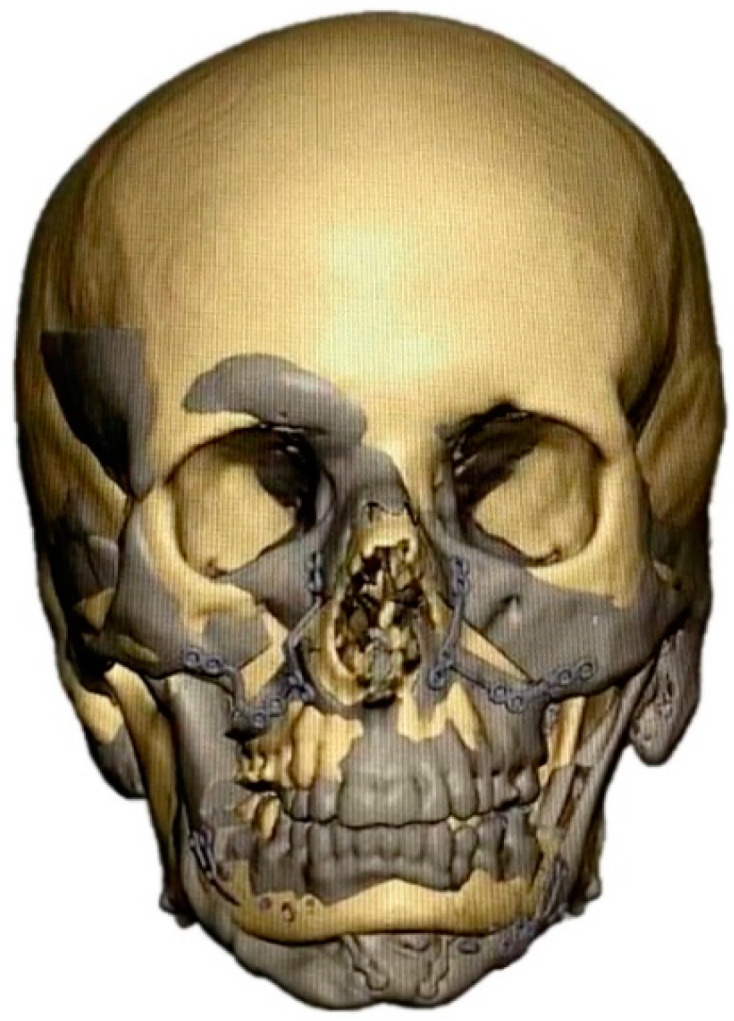
Superimposition of patient skull and standard skull (after adjustment of size). Superimposed and color-marked are the planned postoperative hard tissue contours (gray) and the standard skull (beige).

**Figure 5:**
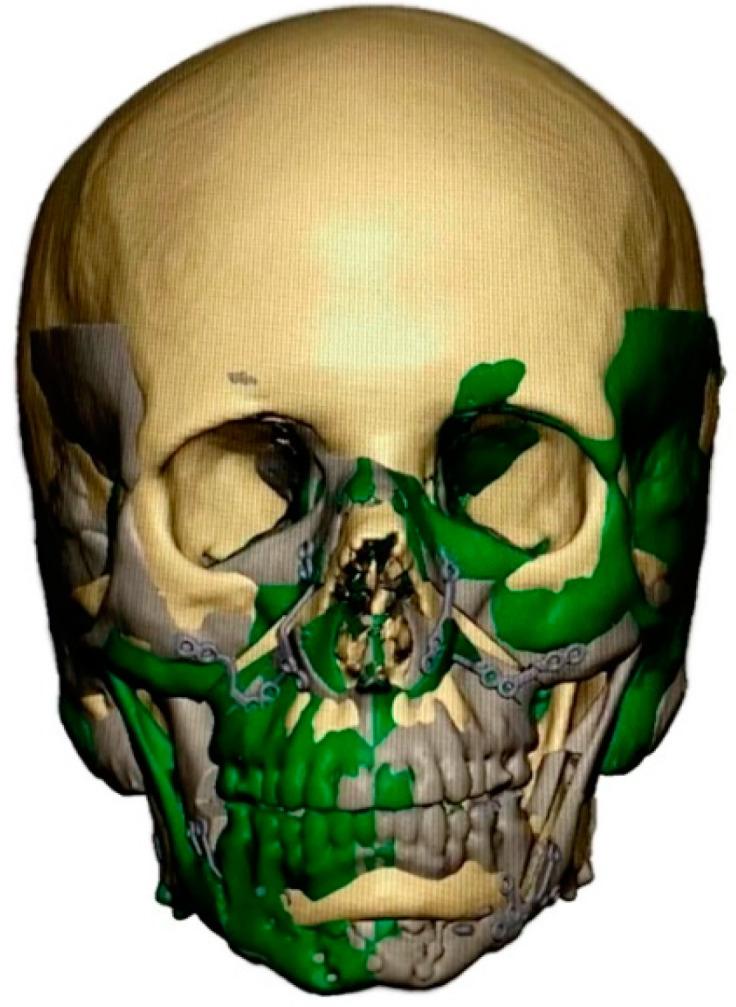
Simultaneous superimposition and mirroring strategy. Superimposed and color-marked are the planned postoperative hard tissue contours (gray), the hard tissue contours mirrored along the median sagittal axis (green), and the standard skull (beige).

In patients with additional PEEK bone augmentation (groups 4–5), PEEK implants were fabricated in a second virtual planning step using a mirroring and/or superimposition strategy (Figure 3, Figure 4 and Figure 5) in a web-based meeting between the surgeon and the manufacturer’s (Synthes GmbH, Oberdorf, Switzerland) engineers. The software used was PROPLAN CMF (Version 3.0.1) (Materialise GmbH, Munich, Germany).

### 2.3. Surgical Approach

In all groups, patients received bimaxillary surgery in a first treatment step (Figure 1 and Figure 2). The maxillomandibular complex was fully mobilized with a conventional Le Fort I osteotomy and a mandibular bilateral sagittal split osteotomy [38]. Planning and execution of the operations were performed based on a lateral and frontal cephalometric analysis using CBCT investigations, subsequent model surgery and split based positioning of the jaws. PEEK bone augmentation was performed in two groups: (i) patients with laterognathy (group 4) received mirroring of the unaffected side (Figure 5), and (ii) patients with malformations (group 5) received superimposition of a (relatively matched) standard skull on the diseased skull (Figure 3 and Figure 4). PEEK implants were selected individually depending on the defect present. Patients (groups 4 and 5) received between one and six PEEK implants, with each site of the viscerocranium having been augmented individually (mandibular, maxillary, temporal, zygomatic, periorbital). Nine months after orthognathic surgery, titanium plates were removed in all patients. PEEK implants were left in place. All patients received orthodontic treatment both before and after the surgical procedure.

### 2.4. Collection of Landmarks and Axes

#### 2.4.1. Photographic Analysis

Standardized en face photographs were taken preoperatively and at least nine months postoperatively (i.e., after removal of the metal) by the same experienced photographer. Photographs were taken with a camera located 1.5 m from the patient’s face. Indirect anthropometric measurements were performed on 60 digital photographs using ImageJ 1.52k (National Institutes of Health, Bethesda, MD, USA) by an evaluator without knowledge of the patients’ medical histories.

**Figure 6:**
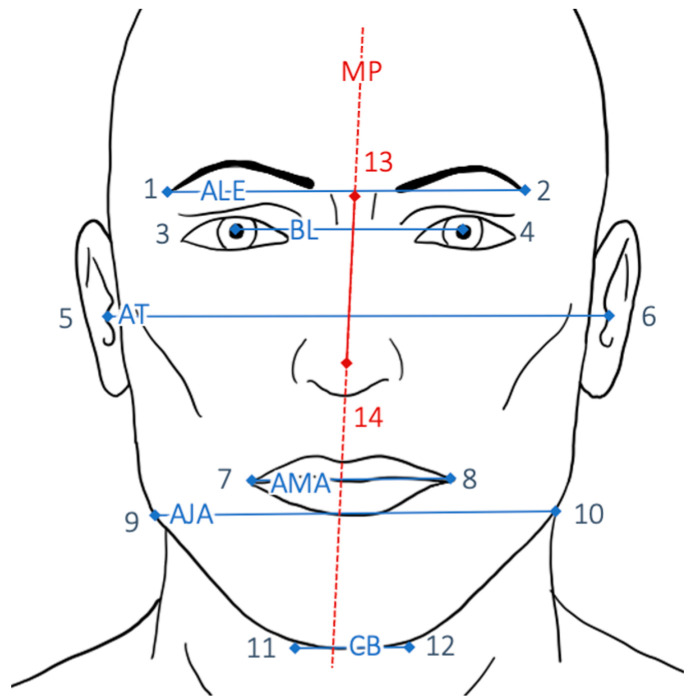
Photographic landmarks and axes as described in Table 2. Ideally the vertical midfacial plane (MP) should be perpendicular to all six axes axis of lateral eyebrows (ALE), bipupillary line (BL), axis of tragi (AT), axis of mouth angle (AMA), axis of jaw angles (AJA), and chin base (CB). Midfacial plane is defined as the axis between the glabella (13) and the tip of the nose (14) and represents a continuous perpendicular to the horizontal facial planes.

For angular measurements, 14 anthropometric landmarks were defined (Figure 6) and measured. The X- and Y-coordinates of the landmarks were transferred in the form of data sets. Six axes—ALE, BL, AT, AMA, AJA, and CB (Table 2)—were derived from the data set, and the respective angle to the perpendicular midfacial plane was formed. The deviation of each angle from the ideal angle to MP (90°) was recorded. Then, the difference between pre- and postoperative deviations was determined. A statistical analysis for each group was performed to obtain descriptive statistics.

#### 2.4.2. Radiological Analysis

CBCT images were taken preoperatively and at least nine months postoperatively (i.e., after removement of the metal) by KAVO 3D exam-vision (extended field view version 17 × 23 cm). Indirect anthropometric measurements were performed on 60 CBCTs using OsiriX 12.0 (Pixmeo SARL, Bernex, Switzerland) by an observer blinded to patients’ medical history. The view was set to maximum intensity projection (MIP) to ensure the best view on all regions of interest as a posteroanterior cephalometric radiograph. X- and Y-coordinates of the landmarks were transferred as a data record. Radiological analysis was performed to investigate changes of hard tissue, including skeletal as well as PEEK bone augmentation structures. Both angular and linear distance measurements were performed as explained below.

**Figure 7:**
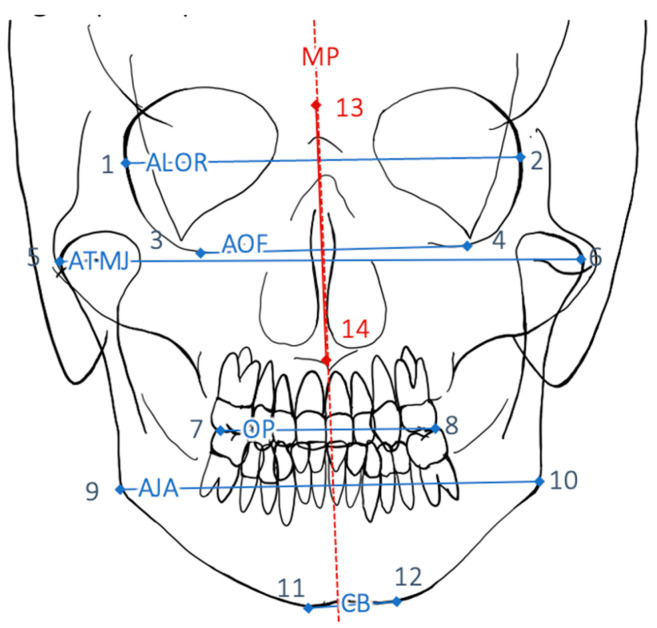
Radiological landmarks and axes as described in Table 3. Ideally the vertical (MP) should be perpendicular to all six axes axis of lateral orbital walls (ALOR), axis of orbital floors (AOF), axis of temporomandibular joints (ATMJ), occlusal plane (OP), axis of jaw angles (AJA), and chin base (CB).

For angular measurement, the axes axis of lateral orbital walls (ALOR), axis of orbital floors (AOF), axis of temporomandibular joints (ATMJ), occlusal plane (OP), axis of jaw angles (AJA), and chin base (CB) (Figure 7 and Table 3) were derived from the data set and the respective angle to the midfacial plane was formed. The deviation of each angle from the ideal angle to MP (ideal angle: 90°) was recorded and then the difference between the preoperative and the postoperative deviations was determined. A statistical evaluation of each group was performed to obtain descriptive statistics.

**Figure 8:**
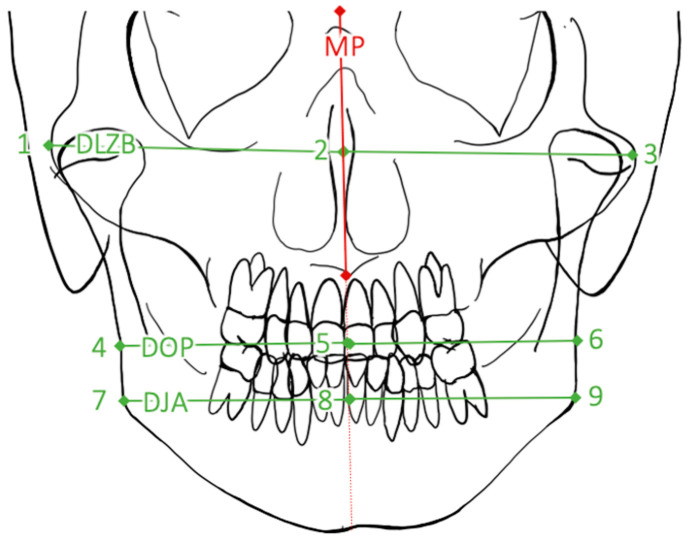
Radiological distances as described in Table 4. These were divided into a left and right part by midfacial plane (red). Ideally the left part of the distance should be as long as the right part.

For linear distance measurement, the distances between midfacial plane (MP) and farthest extension of the right and left facial side were obtained on three facial levels (distance on level of zygomatic bones (DLZB), distance on level of occlusal plane (DOP), and distance on level of jaw angles (DJA)) (Figure 8 and Table 4). The distances of each level to MP were recorded. Then, the difference (ideally: 0 mm) between the preoperative and the postoperative distances was determined. A statistical evaluation of each group was performed to obtain descriptive statistics.

### 2.5. Analyses of Symmetry

The previously obtained data from the photographic and radiological analyses were processed and statistically analyzed using IBM^®^ SPSS^®^ Statistics (version 27, SPSS Inc., Chicago, IL, USA) and Microsoft^®^ Excel (version 16.45, Microsoft, Redmond, WA, USA).

Photographic axes ALE, BL, AT, AMA, AJA, and CB (Figure 6) as well as radiological axes ALOR, AOF, ATMJ, OP, AJA, and CB (Figure 7) were derived from the data set and the respective angle to the midfacial plane was formed, both prior to surgery and after surgery. The deviation of each angle from the ideal angle to MP (ideal angle: 90°) was recorded.

Radiological distances on levels DLZB, DOP, and DJA were measured for linear distance investigation. Left and right side of each face and level were compared and discrepancies between the left to the right distance were determined.

### 2.6. Statistical Analyses

A power analysis was performed using G*Power (version 3.1.9.6, Heinrich Heine University, Düsseldorf, Germany).

Pearson’s correlation analysis was performed to evaluate the correlation between asymmetry pre-op and extent of normalization post-op. Statistical analysis was performed at a significance level of *p* < 0.05 with IBM^®^ SPSS^®^ Statistics (version 27). The difference between pure conventional orthognathic surgery and orthognathic surgery plus simultaneous PEEK bone augmentation was investigated by an independent sample test with *n* = 58 values. First, Levene’s test for equality of variances was applied. After that, an unpaired two-tailed *t*-test was performed with a level of significance of *p* < 0.05.

Furthermore, a multivariable regression analysis was performed to evaluate the outcome (soft tissue deviation from ideal) and its dependency by covariates (whether or not PEEK augmentation was performed, baseline soft tissue deviation from ideal, postoperative bony deviation from ideal).

### 2.7. Error Study

Eight cases were randomly selected to evaluate the reliability of the measurements. Measurements (X- and Y-coordinates) were recorded and remeasured two times. All data were compared by a paired t-test and presented no significant difference at remeasuring.

## 3. Results

### 3.1. Comparing Angular Deviations Pre- versus Postoperatively

Angular deviations could be reduced in all four laterognathic groups (groups 2, 3, 4, and 5), while the non-laterognathic group (group 1) did not receive major changes.

### 3.2. Angular Normalization in Soft Tissue

**Figure 9:**
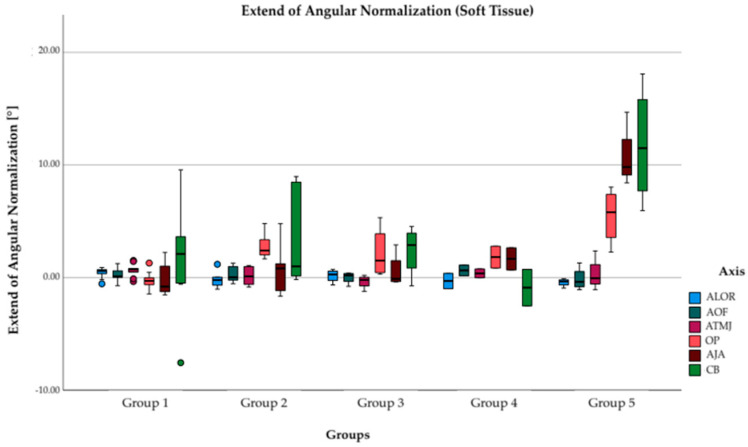
Extent of angular normalization in soft tissue after complete surgical treatment. Columns representing mean symmetry changes in degrees of soft tissue in each group. Mean values of preoperative and postoperative symmetry deviations were subtracted resulting in positive values for improvement and deterioration of symmetry. Both axis of lateral eyebrows (ALE) (light blue), bipupillary line (BL) (dark green) as well as axis of tragus (AT) (violet) show only few changes in all groups. Axis of mouth angle (AMA) (orange), axis of jaw angle AJA (dark violet), and chin base CB (green) show improvements in facial symmetry in group 2, 3, 4, and 5. However, largest improvements could be investigated in group 5.

The difference between the angular deviation in soft tissue before and after surgical treatment can be seen in Figure 9. Both axes lateral eyebrows (ALE), bipupillary lines (BL) as well as tragus (AT) show only little changes. Axis of mouth angle (AMA), axis of jaw angle (AJA), and chin base (CB) show improvements in facial symmetry in group 2, 3, 4, and 5. (AMA) were improved by 1.8–2.8° (groups 2–4). However, the greatest improvements in soft tissue after surgical treatment were achieved in group 5, where, while the axis of mouth angle could be improved by 5.5°, even larger improvements in symmetry could be seen in jaw angle (10.7°) and chin base (11.8°).

### 3.3. Angular Normalization in Hard Tissue

**Figure 10:**
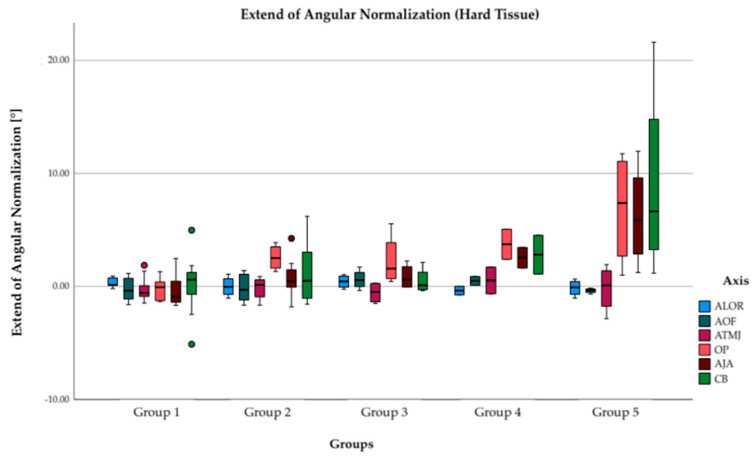
Extent of angular normalization in hard tissue after complete surgical treatment. Each column demonstrates mean values of angular changes in degrees of hard tissue after surgical treatment in each group. Mean values of preoperative and postoperative symmetry deviations were subtracted resulting in positive values for improvement and deterioration of symmetry. Both axis of lateral orbital rim (ALOR) (light blue), axis of orbital floor (AOF) (dark green) as well as axis of temporomandibular joints (ATMJ) (purple) show only few changes. Both axes occlusal plane (OP) (orange), axis of jaw angles (AJA) (dark purple), as well as chin base (CB) (green) show improvements in facial symmetry in group 2, 3, 4, and 5. However, largest improvements could be investigated in group 5.

The difference between the angular deviation in the hard tissue before and after surgical treatment can be seen in Figure 10. Both axes lateral orbital walls (ALOR), orbital floors (AOF) as well as temporomandibular joints (ATMJ) show only little changes. However, improvements in facial symmetry are seen in occlusal plane (OP) (2.3–3.7°), axis of jaw angles (AJA) (0.8–2.5°), as well as chin base (CB) (0.5–2.8°) in groups 2, 3, and 4. However, the greatest improvements in symmetry were seen in group 5, where major enhancements of symmetry could be seen in occlusal plane (6.9°), axis of jaw angles (6.2°), and chin base (9.0°).

### 3.4. Comparing Linear Distance Discrepancy Pre- versus Post-Op

**Figure 11:**
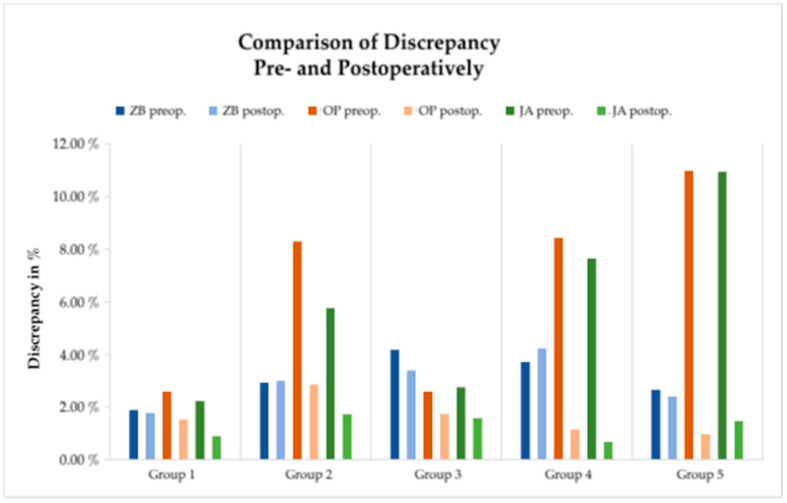
Comparison of pre- and postoperative discrepancy. Mean percentage discrepancy of facial left side versus right side at different levels (ZB = zygomatic bone (blue), OP = occlusal plane (red), and JA = jaw angle (green)) pre- and postoperative. Deviations on the level of zygomatic bone (blue) stay the same, while each group shows less deviation on the levels of occlusal plane (red) and jaw angle (green) after surgical treatment. Groups 4 and 5, in particular, show massively decreased discrepancy after surgical treatment.

**Figure 12:**
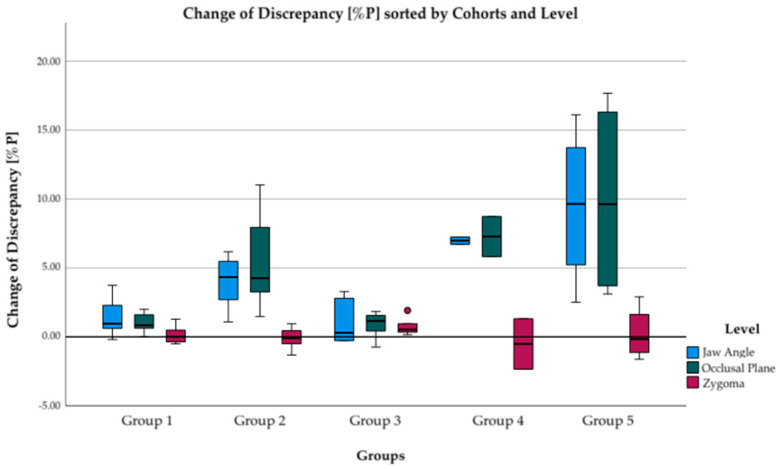
Boxplots displaying change of discrepancy in percentage points subdivided into groups 1–5. Positive values are improvements in symmetry, while negative values mean a deterioration in symmetry. Improvements occur at the levels of jaw angle (blue) and occlusal plane (green). However, there are no changes in the discrepancy at the level of zygoma (red) in any of the groups.

The difference between linear distance discrepancy in hard tissue before and after surgical treatment can be seen in Figure 11. On zygomatic bone level, pre- and post-op discrepancies differ only slightly. However, in occlusal plane and in jaw angle level, the postoperative discrepancy values are much lower than the preoperative values. This reduction in discrepancy is most pronounced in groups 4 and 5, with a relative change of discrepancy of 86 to 91% (Figure 12).

### 3.5. Correlation between Asymmetry and Extent of Normalization

**Figure 13:**
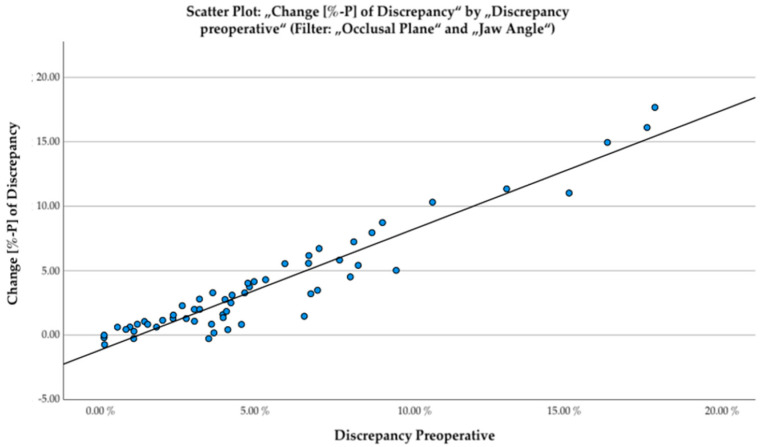
Scatter plot displaying reduction of discrepancy in percentage points (change of discrepancy) vs. amount of preoperative asymmetry (discrepancy pre-op).

At the levels of *occlusal plane* and *jaw angle* there is high correlation between the extent of asymmetry before surgical treatment and the change in discrepancy gained after surgical intervention (Figure 13). Both variables showed a high positive correlation (r (56) = 0.95; *p* < 0.01).

### 3.6. Multivariable Regression

Multivariable regression yielded a negative coefficient value of −0.82 when additional PEEK augmentation was used. The coefficient on baseline soft tissue deviation from ideal was 0.18. For postoperative bony deviation from ideal, the coefficient was negative (−0.15). The results are not statistically significant (*p* > 0.05) (see Table 5).

### 3.7. Difference of Normalization between Pure Conventional Surgery and Simultaneous PEEK Bone Augmentation

**Figure 14:**
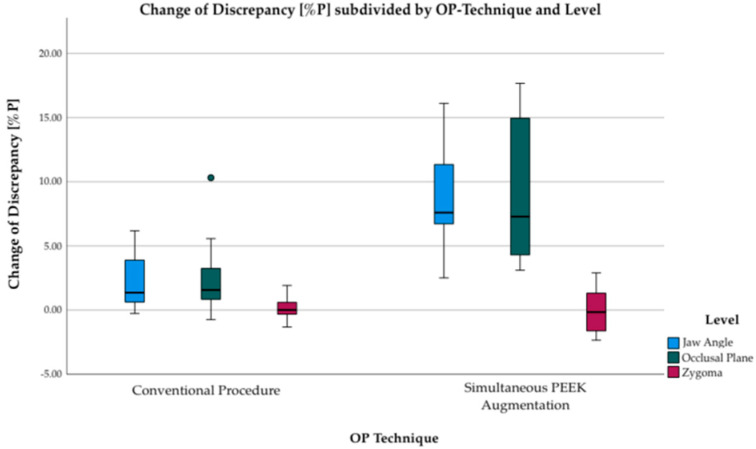
Boxplots displaying change of discrepancy in percentage points subdivided into orthognathic surgery only (cumulative groups 1, 2, and 3) and orthognathic surgery with additional PEEK bone augmentation (cumulative groups 4 and 5). Positive values are improvements in symmetry, while negative values mean a deterioration in symmetry. Improvements occur at the levels of jaw angle (blue) and occlusal plane (green) with mean 2.4 percentage points in orthognathic surgery only and mean 8.9 percentage points in orthognathic surgery with additional PEEK bone augmentation. However, there are no changes in the discrepancy at the level of zygoma (red) in any of the groups.

Both surgical procedures, pure conventional orthognathic surgery as well as orthognathic surgery with simultaneous PEEK bone augmentation, reduced discrepancies between the left and the right side of the face. Reduction of discrepancies was observed at the levels of jaw angle and occlusal plane, while the values at the level of zygoma remained unchanged in both procedures. Pure conventional surgery reduced discrepancies at the level of jaw angle and occlusal plane by an average of 2.4 percentage points (%P). Even more, orthognathic surgery with simultaneous PEEK bone augmentation reduced discrepancies by an average of 8.9%P, resulting in a significant difference of 6.5%P (3.2–9.8) (*p* = 0.001). Figure 14 shows the changes of discrepancy in a boxplot diagram, subdivided by surgical procedure (whether with or without simultaneous PEEK bone augmentation).

### 3.8. Outcome after Surgical Treatment

**Figure 15:**
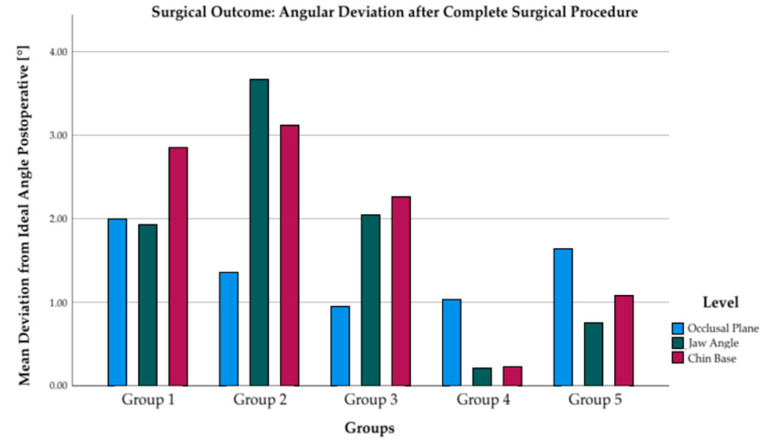
Angular deviation from ideal angle to midfacial plane (90°) after complete surgical treatment.

**Figure 16:**
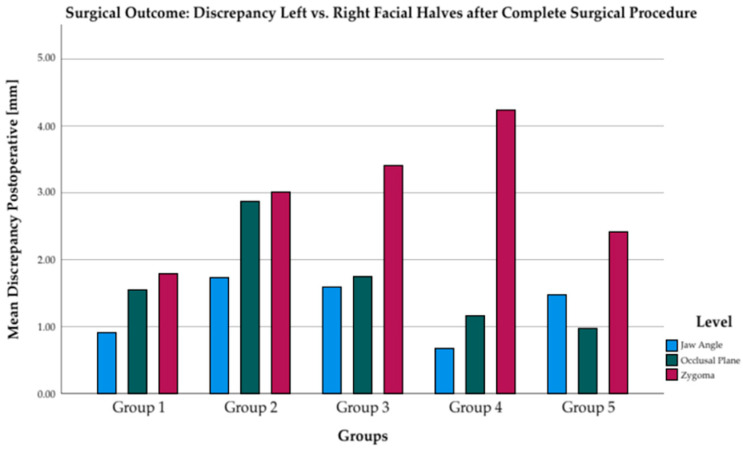
Linear distance discrepancy of left vs. right facial halves subdivided by all levels after surgical treatment in all five groups.

The outcome after completed surgical treatment, subdivided into the individual groups, is shown in Figure 15 and Figure 16. The mean angular deviations are between 1–3°. Groups 4 and 5 show the lowest angular deviation at the jaw angle and chin base levels.

The mean differences between the right and left sides of the face after completed surgical treatment are 1–4 mm. At the level of zygoma, the greatest discrepancies can be seen between the left and right sides of the face. The discrepancies at the level of the jaw angle and occlusal plane are less than 2 mm in all groups but are consistently smallest in groups 4 and 5.

Intraoral exposure of PEEK implants occurred in two patients. This complication was detected during follow-up visits and corrected by grinding the PEEK implants in situ. Besides, no other complications of PEEK implants, such as local inflammation or material fractures, were noted.

## 4. Discussion

The results of the present study show that all laterognathic patients benefited from orthognathic surgery with improved postoperative symmetry. With the help of PEEK bone augmentation almost ideal facial angles (less than 2° deviation from perfect mathematical symmetry) could be achieved. The minimal deviations found in the present study correspond well to the deviations of up to 1.3 ± 0.3° that Song et al. (2007) [26] defined as symmetrical in healthy (non-laterognathic) patients. According to other scientific studies, this deviation is considered normal and harmonious [37].

The results of this study demonstrate the significant improvement of facial symmetry by simultaneous PSI-based orthognathic surgery and PEEK bone augmentation surgery for craniofacial malformations. In addition to facial skeletal symmetry, soft tissue symmetry could be established.

### 4.1. Results

Improvements in symmetry were observed in all four laterognathic groups (groups 2–5), whereas the non-laterognathic group (group 1 = control group) received no benefits in frontal symmetry. This can be explained by the fact that orthognathic surgery in non-laterognathic patients aims to improve sagittal midface structures but primarily has no effect on facial structures in frontal view [39].

In this study, hard tissue improvements were only seen in the occlusal plane (OP), the axis of jaw angles (AJA) and the chin base (CB) whereas changes at the midface level (orbit, zygoma, tragus) were not detected. This can be explained by the fact that orthognathic surgery comes with significant bony changes in the jaws while the midface remains unaffected. Simultaneous augmentation with PEEK implants showed no effect on hard tissue symmetry, but enhanced the soft tissue outcome [40].

The analysis of soft tissue symmetry improvements showed similar amendments as in the hard tissue analysis. Thus, it could be shown that with the help of simultaneous orthognathic and augmentative surgery not only hard but also soft tissue symmetry is be established.

However, these data primarily allow a statement for symmetry improvements in the lower half of the face, even when augmentations were performed in the midface. This is surprising, since it was expected that augmentations in the midface would also result in significant improvements at the same level (ALE, BL, AT). One reason could be that despite PEEK augmentation of the bony orbit the lateral eyebrows (ALE) remain asymmetrical as skin appendages even if the underlying soft tissue has become more symmetrical. The bipupillary line (BL) also is not changed by augmentation of the orbital rims, but only by alteration of the orbital walls. However, this was not intended in the context of the surgery performed here. Likewise, no relevant changes are detected at the tragus (AT), since no orthognathic or augmentative changes were performed here. Accordingly, improvements in midface symmetry are to be expected, but these may not have been sufficiently considered in the present method section and therefore cannot be clearly demonstrated.

While all four laterognathia groups (groups 2–5) benefited from orthognathic surgery in these midfacial axes, greater improvements were achieved in syndromic laterognathia patients (group 5) treated by orthognathic surgery with simultaneous PEEK bone augmentation. This indicates a therapeutic advantage with PEEK bone augmentation in severe facial malformations. However, it should be noted that this patient population also includes patients with branchial arch diseases associated with hemifacial microsomia. Therapy of these patients inevitably leads to significant improvements in soft tissue symmetry. Consequently, this group also shows best results.

Even in patients with very severe laterognathic conditions, both non-syndromic (group 4) and non-syndromic (group 5), harmonious symmetry could be achieved by the presented treatment concept of simultaneous orthognathic and augmentative surgery. At discharge form the hospital, patients had a symmetrical face with a deviation of approximately 2° and 2 mm from ideal mathematical symmetry (Figure 15 and Figure 16). According to scientific studies, this deviation is considered normal and harmonious. [37]

#### 4.1.1. Correlation between Severity of Disease and Extent of Normalization

Disease severity was associated with greater improvement in symmetry after complete surgery (Figure 13). On the one hand, this is because greater asymmetry provides more room for improvement. On the other hand, according to our study design, patients with more severe deformities were more likely to be treated with additional PEEK bone augmentation and thus benefited from the modern surgical procedure.

#### 4.1.2. Multivariable Regression

By using a multivariable regression analysis, the influence of the different independent coefficients (PEEK augmentation, baseline soft tissue deviation from ideal and postoperative bony deviation from ideal, postoperative soft tissue deviation from ideal) could be demonstrated, indicating the effect of performing PEEK augmentation on postoperative soft tissue symmetry. None of the results were statistically significant. However, they showed a tendency towards a positive effect of PEEK augmentation on soft tissue symmetry. Furthermore, they reveal a positive effect of baseline soft tissue deviation from ideal on postoperative soft tissue symmetry—admittedly a very small one (0.18). This is intuitive, as greater preoperative asymmetry is well matched with greater postoperative asymmetry.

Interestingly, postoperative bony deviation had a negative effect on postoperative asymmetry (−0.15), leading to lower postoperative asymmetry. This might be explained by observations in orthognathic surgery showing that skeletal conversion causes only 76% soft tissue conversion [41,42,43].

#### 4.1.3. Comparison between Conventional Orthognathic Procedure and Simultaneous PEEK Bone Augmentation

A comparison of both procedures, pure conventional orthognathic surgery and orthognathic surgery with simultaneous PEEK bone augmentation, shows the advantage of simultaneous PEEK bone augmentation. Angular and linear improvements in symmetry are significantly greater with the use of PEEK bone augmentation. This demonstrates the advantages of PEEK bone augmentation as an extension of conventional orthognathic surgery. This result is also consistent with other study results that investigated the advantage of PEEK bone augmentation surgery in general maxillofacial surgery [11].

#### 4.1.4. Complications of PEEK Implants

In this study, two of the eight patients who underwent PEEK augmentation suffered from exposure to the implant. No other complications occurred. This corresponds to a complication rate of 25%. In a meta-study examining complications following PEEK implants for cranioplasty, the complication rate was 15.3% (*n* = 183) [44]. This suggests a possibly higher complication rate with the combined orthognathic and augmentation procedure presented in our study. However, due to the small number of subjects in the present study (*n* = 8), a statistically significant comparison cannot be made until subsequent studies with a larger patient size have been conducted. It should be noted, however, that these complications could be corrected by a short re-operation in local anesthesia during follow-up and had no further influence on the surgical outcome.

### 4.2. Validity and Reliability

The combination of photographic and cephalometric radiographical analyses provides a reliable method for the detection of facial asymmetries [45]. En face photographs provide reliable information on facial asymmetry [26] and CBCT provide reliable information in detection of craniofacial asymmetry [46].

All processes in this study were highly standardized. While all surgical procedures were performed by the same surgeon, all measurements were conducted by the same examiner. This leads to a high internal validity, that is also demonstrated by the consistent measured values for parameters that are not altered by the surgical procedure. Thus, both the axis between the two lateral orbital rims (ALOR) and the axis of orbital floor (AOF) are consistent pre- and postoperatively. These axes indicate the vertical alignment of the neurocranium and are not affected by midfacial surgery. The angles between ALOR/midface plane (MP) and AOF/MP average 0.7° in all five groups. This highlights the high level of internal validity and reliability.

However, external validity, which allows for transferability to a larger patient population, is limited in this study by the single-surgeon principle. Further studies involving other surgical centers and surgeons as well as more patients may result in an even higher level of external validity.

### 4.3. Limitations

Due to the rarity of severe craniofacial malformations, only a limited number of patients were included in this study. Only mature patients were treated (after completion of their growth). Therefore, a subdivision by age and sex could not be made. However, many studies have shown that neither age [47] nor sex [48,49] has an influence on the degree of asymmetry. Therefore, the inhomogeneity of the patient population is not a disadvantage in this study.

Facial asymmetry can be determined by midfacial symmetry measurements, as shown in the present study. However, some researchers compare pairwise measurements of bilateral facial features to obtain information about the overall 3D nature of asymmetry [50,51,52]. In this study, we focused on 2D imaging techniques by using preoperative and postoperative photographs and posteroanterior analyses of 3D cephalometric radiographs (CBCT scans) in a medio-lateral spread. This method ensures unambiguous assignment of measurements in the coronary direction and reduces confounding effects due to sagittal asymmetries. However, further studies should also investigate the effects of orthognathic surgery with additional PEEK bone augmentation on sagittal asymmetries.

In clinical practice, it is noticeable that the classification of patients with jaw malocclusions is very complex. The generally applicable classification system according to International Classification of Diseases coding (ICD) [53] only provides a very superficial classification. This makes it difficult to assign patients to cohorts and different scientists and clinicians use heterogeneous classifications. Therefore, classification and comparability to other studies is limited. For future studies, the introduction of a new classification system could be beneficial, as suggested by Gateno, Alfi [54].

### 4.4. Clinical Considerations

CAD/CAM-based augmentation procedures provide surgeons with a modern and excellent tool to safely plan and perform surgery even in complex craniofacial situations. Such a surgical method, including PSI-based orthognathic surgery and PEEK bone augmentation, requires careful, time-consuming planning and provides further costs [18]. However, this is compensated by the significantly reduced operation time. In addition, the simultaneous approach described in this study invalidates these disadvantages, as the single-stage approach can significantly reduce both planning and surgical time.

Bone augmentation can be performed using silicone, titanium or PEEK [33,55]. The advantages of PEEK bone implants are their light weight, radio translucency [29] and lack of impact on imaging modalities such as CT or MRI [56].

Furthermore, despite all the advantages of the combined PSI-based orthognathic procedure with the additional placement of PEEK bone augmentations, the negative aspects should not be ignored. In particular, the insertion of foreign material carries a risk of infection [57]. However, this risk can be countered by appropriate antibiotic shielding measures [58]. In addition to infection, exposure of the PEEK bone implants or recurrences may occur, especially with larger augmentations [59]. In case reports on patient-specific PEEK bone augmentations of the mandible Arcas Pons, Vendrell [60] noted that no infections, intolerances to the PEEK bone implants, or exposures occurred in long-term follow-up after the initial healing process. This is consistent with our outcome. Furthermore, the effects on patient safety and satisfaction should be evaluated in large-scale studies to objectify further benefits of this single-stage surgical approach presented here.

## 5. Conclusions

Simultaneous PSI-based orthognathic surgery with PEEK bone augmentation provides significant improvement of facial symmetry in patients with craniofacial malformations. In addition to facial skeletal symmetry, soft tissue symmetry was established. The digital workflow, including virtual surgical planning, resulted in symmetry improvement in all patients.

## Figures and Tables

**Table 1:** Subdivision of patients into five groups.

#	Laterognathy	Syndrome	Surgical Procedure	*n*
1	non-laterognathic	non-syndromic	orthognathic surgery	10
2	laterognathic	non-syndromic	orthognathic surgery	8
3	non-laterognathic	syndromic	orthognathic surgery	4
4	laterognathic	non-syndromic	orthognathic surgery + augmentation	4
5	laterognathic	syndromic	orthognathic surgery + augmentation	4

**Table 2:** Axes between two defined points (see Figure 6). Six angles between each axis and the midfacial plane are determined.

Points	Abbreviation	Description
1–2	ALE	Axis of lateral eyebrows
3–4	BL	Bipupillary line
5–6	AT	Axis of tragi
7–8	AMA	Axis of mouth angle
9–10	AJA	Axis of jaw angles
11–12	CB	Chin baseline
13–14	MP	Midfacial plane

**Table 3:** Axes between two defined points (see Figure 1). Six angles between each axis and the midfacial plane are determined.

Points	Abbreviation	Description
1–2	ALOR	Axis of lateral orbital walls
3–4	AOF	Axis of orbital floors
5–6	ATMJ	Axis of temporomandibular joints
7–8	OP	Occlusal plane
9–10	AJA	Axis of jaw angles
11–12	CB	Chin baseline
13–14	MP	Midfacial plane

**Table 4:** Distances between two defined points. Division of each distance results in six linear distance measurements in total.

Points	Abbreviation	Description
1–2; 2–3	DLZB	Right and left distances of lat. zygomatic bone
4–5; 5–6	DOP	Right and left distances of occlusal plane
7–8; 8–9	DJA	Right and left distances of jaw angle

**Table 5:** Multivariable regression analysis of coefficients. Dependent variable: soft tissue deviation from ideal.

	Unstd. B	Coeff. Std. Error	Std. Coeff. Beta	*t*	Sig.	95 % Conf. Int.	
						Lower Bound	Upper Bound
**(Constant)**	−0.285	0.295		−0.966	0.345	−0.900	0.330
**PEEK Augmentation yes (1) or no (0)**	−0.817	0.630	−0.271	−1.296	0.210	−2.131	0.498
**Baseline Soft Tissue Deviation from Ideal**	0.176	0.093	0.395	1.888	0.074	−0.018	0.370
**Postoperative Bony Deviation from Ideal**	−0.146	0.132	−0.220	−1.106	0.282	−0.422	0.130

## Data Availability

The data presented in this study are available on request from the corresponding author. The data are not publicly available due to the decision of the ethics committee.
